# Supplementary material for: Characterising Carbon Monoxide Household Exposure and Health Impacts in High- and Middle-Income Countries—A Rapid Literature Review, 2010–2024
Source: Int J Environ Res Public Health. 2025 Jan 15;22(1):110. doi: 10.3390/ijerph22010110 (PMC11764952; doi:10.3390/ijerph22010110)
Supplement: Supplementary file 1 [file ijerph-22-00110-s001.zip › Supplementary_material_1.pdf]

S1 FILE – SUPPLEMENTARY MATERIAL

S1 – Search strategies

	**Ovid Medline ALL**		**Ovid Embase**
1	carbon monoxide.tw,kf.	1	carbon monoxide.tw,kf.
2	Carbon Monoxide/	2	Carbon Monoxide/
3	1 or 2	3	1 or 2
4	indoor*.tw,kf.	4	indoor*.tw,kf.
5	(house* or dwelling* or home*).tw,kf.	5	(house* or dwelling* or home*).tw,kf.
6	domestic.tw,kf.	6	domestic.tw,kf.
7	(gas adj (boiler* or oven* or cooker* or heater* or fire* or stove*)).tw,kf.	7	(gas adj (boiler* or oven* or cooker* or heater* or fire* or stove*)).tw,kf.
8	(wood fire* or (wood adj2 stove*)).tw,kf.	8	(wood fire* or (wood adj2 stove*)).tw,kf.
9	paraffin heater*.tw,kf.	9	paraffin heater*.tw,kf.
10	coal fire*.tw,kf.	10	coal fire*.tw,kf.
11	generator*.tw,kf.	11	generator*.tw,kf.
12	(cooking stove* or cooking appliance*).tw,kf.	12	(cooking stove* or cooking appliance*).tw,kf.
13	heating appliance*.tw,kf.	13	heating appliance*.tw,kf.
14	kitchen*.tw,kf.	14	kitchen*.tw,kf.
15	(room or room*).tw,kf.	15	(room or room*).tw,kf.
16	residential.tw,kf.	16	residential.tw,kf.
17	Air Pollution, Indoor/	17	indoor air pollution/
18	exp Home Environment/	18	home environment/
19	Housing/	19	Housing/
20	Residential Facilities/	20	boiler/

21	4 or 5 or 6 or 7 or 8 or 9 or 10 or 11 or 12 or 13 or 14 or 15 or 16 or 17 or 18 or 19 or 20	21	4 or 5 or 6 or 7 or 8 or 9 or 10 or 11 or 12 or 13 or 14 or 15 or 16 or 17 or 18 or 19 or 20
22	model*.tw,kf.	22	model*.tw,kf.
23	(measure* or detect* or assess*).tw,kf.	23	(measure* or detect* or assess*).tw,kf.
24	monitor*.tw,kf.	24	monitor*.tw,kf.
25	biomarker*.tw,kf.	25	biomarker*.tw,kf.
26	(questionnaire* or survey*).tw,kf.	26	(questionnaire* or survey*).tw,kf.
27	COHb.tw,kf.	27	COHb.tw,kf.
28	(observation or observations).tw,kf.	28	(observation or observations).tw,kf.
29	(alarm or alarms).tw,kf.	29	(alarm or alarms).tw,kf.
30	carboxyhemoglobin.tw,kf.	30	carboxyhemoglobin.tw,kf.
31	methodolog*.tw,kf.	31	methodolog*.tw,kf.
32	misclassification.tw,kf.	32	misclassification.tw,kf.
33	metrics.tw,kf.	33	metrics.tw,kf.
34	Environmental Monitoring/	34	Environmental Monitoring/
35	Data Collection/	35	information processing/
36	Environmental Biomarkers/	36	environmental marker/
37	"Surveys and Questionnaires"/	37	questionnaire/
38	Carboxyhemoglobin/	38	Carboxyhemoglobin/
39	22 or 23 or 24 or 25 or 26 or 27 or 28 or 29 or 30 or 31 or 32 or 33 or 34 or 35 or 36 or 37 or 38	39	model/
40	3 and 21 and 39	40	22 or 23 or 24 or 25 or 26 or 27 or 28 or 29 or 30 or 31 or 32 or 33 or 34 or 35 or 36 or 37 or 38 or 39
41	4 or 17	41	3 and 21 and 40
42	exposure*.tw,kf.	42	4 or 17
43	3 and 41 and 42	43	exposure*.tw,kf.
44	40 or 43	44	3 and 42 and 43
45	biomass.tw,kf.	45	41 or 44
46	Biomass/	46	biomass.tw,kf.
47	exp animals/ not humans.sh.	47	Biomass/
48	45 or 46 or 47	48	(exp animal/ or nonhuman/) not exp human/
49	44 not 48	49	46 or 47 or 48
		50	45 not 49
		51	limit 50 to conference abstracts
		52	50 not 51

	**Web of Science (WoS)**	**Google scholar**

1	AK=("environmental monitoring" or questionnaire* or "data collection" or "environmental biomarker*" or carboxyhemoglobin)	allintitle: carbon monoxide indoors -biomass.<br>allintitle: carbon monoxide models -biomass<br>allintitle: carbon monoxide monitoring -biomass
2	TS=(methodolog* or misclassification or metrics)	
3	TS=(alarm or alarms)	
4	TS=(questionnaire* or survey* or observation or observations)	
5	TS=(biomarker* or COHb or carboxyhemoglobin)	
6	TS=(model* or measure* or detect* or assess* or monitor*)	
7	#1 OR #2 OR #3 OR #4 OR #5 OR #6	
8	TS=(indoor* or house* or home* or dwelling*)	
9	TS=(domestic or "gas boiler*" or "gas oven*" or "gas cooker*" or "gas heater*" or "gas fire*" or "gas stove*")	
10	TS=(wood fire* or "wood stove*" or "wood burning stove*")	
11	TS=("paraffin heater*" or "coal fire" or "coal fires" or generator*)	
12	TS=("cooking stove*" or "cooking appliance*" or "heating appliance*")	
13	TS=(kitchen* or room or room* or residential)	
14	AK=(indoor air pollution)	
15	AK=("home environment" or housing)	
16	#8 OR #9 OR #10 OR #11 OR #12 OR #13 OR #14 OR #15	
17	TS=(carbon monoxide)	
18	#16 AND #17	
19	#7 AND #18 Editions: WOS.SCI,WOS.SSCI,WOS.BSCI,WOS.BHCI,WOS.ESCI,WOS.CCR,WOS.IC Timespan: 2010-01-01 to 2023-02-23	
20	TS=(biomass)	
21	TS=(animal* NOT human*)	

22	#21 OR #20	
23	#19 NOT #22	
24	#19 NOT #22 Timespan: 2010-01-01 to 2023-02-24	
25	#19 NOT #22 Editions: WOS.SCI,WOS.SSCI,WOS.BSCI,WOS.BHCI,WOS.ESCI,WOS.CCR,WOS.IC Timespan: 2010-01-01 to 2023-02-24	

S2 – Data extraction template

**Citation**	**Study design**	**Country**	**Study focus**<br>•source<br>•environment<br>•Focus e.g. flow dynamics, ventilation, size etc	**Setting description**<br>• Type e.g. house, flat, bungalow, mobile home<br>• Room(s) that are the focus of the study<br>• Setting characteristics e.g. number of rooms, household size, ventilation, CO sources including cooking and heating system	**Study characteristics**<br>•Number of participants/settings<br>• Location(s)<br>• Study duration, including seasonality<br>Questionnaire on household characteristics etc	**CO monitored/measured**<br>Yes/No	**Further information**<br>•CO measurement device +/- calibration<br>•CO indoor<br>•CO outdoor/ambient<br>•Frequency of CO measured e.g. continuous, time between measurements, specific times<br>•Ventilation/Air exchange measured	**CO modelled**<br>Yes/No	**Brief description of modelling**<br>•including how e.g mathmatical<br>•source<br>•environment<br>•Focus e.g. flow dynamics, venitlation, size etc	**Validated model (e.g. tested with CO data)**<br>Yes/No

**Relevant outcomes**<br>•CO concentrations - mean, median, max, min values<br>•CO concentrations by setting characteristics<br>•CO concentration impacts on people in the household	**Personal level impact**<br>•Health outcomes	**Key findings summarised**	**Relevance**	**Notes**
